# A Short History of Posterior Dynamic Stabilization

**DOI:** 10.1155/2012/629698

**Published:** 2012-12-26

**Authors:** Cengiz Gomleksiz, Mehdi Sasani, Tunc Oktenoglu, Ali Fahir Ozer

**Affiliations:** ^1^Neurosurgery Department, School of Medicine, Mengücek Gazi Training and Research Hospital, Erzincan University, 24000 Erzincan, Turkey; ^2^Neurosurgery Department, American Hospital, 34365 Istanbul, Turkey; ^3^Neurosurgery Department, School of Medicine, Koc University, Rumelifeneri Yolu Sarıyer, 34450 Istanbul, Turkey

## Abstract

Interspinous spacers were developed to treat local deformities such as degenerative spondylolisthesis. To treat patients with chronic instability, posterior pedicle fixation and rod-based dynamic stabilization systems were developed as alternatives to fusion surgeries. Dynamic stabilization is the future of spinal surgery, and in the near future, we will be able to see the development of new devices and surgical techniques to stabilize the spine. It is important to follow the development of these technologies and to gain experience using them. In this paper, we review the literature and discuss the dynamic systems, both past and present, used in the market to treat lumbar degeneration.

## 1. Introduction

Lumbar spine degeneration was first described by Kirkaldy-Willis and Farfan in 1982, using a 3 stage concept: (1) temporal dysfunction, (2) unstable stage, and (3) stabilization [1]. Stage 1 patients may respond to conservative treatments, but stage 2 and stage 3 patients require surgery for stabilization, decompression, and to correct deformities. Although disc degeneration is one reason for chronic lower back pain, the primary reason for back pain is the instability of the lumbar spine [2]. However, lumbar instability is not clearly defined. Kirkaldy-Willis and Farfan defined instability as the clinical status of patients with back problems who, with the least provocation, transition from being mildly symptomatic to experiencing a severe episode [1]. According to Panjabi [3] instability results from the inability to maintain control of the lumbar neutral zone, where spine motion occurs with minimal internal resistance and within normal physiological limits. In this study, instability is defined as the source of pain and abnormal motion. Stokes at al. [4] and Weiler et al. [5] also related abnormal motion to chronic back pain. However, as a definition of instability, abnormal motion does not cause back pain in all cases, such as when abnormal movement is observed radiologically in degenerated discs associated with spondylolisthesis, and pain is not continuous [6]. Therefore, the definition of instability has been updated to include abnormal movements at the joint surface and altered load transmission [2]. Lumbar spinal fusion is a common surgical treatment used in disc degeneration, which is related to chronic lower back pain and other spinal disorders, such as disc herniation, spondylolisthesis, facet arthropathy, and spinal stenosis [7]. Spinal fusion was first described by Albee for the treatment of Pott disease [8] and by Hibbs who performed spinal fusion for the treatment of spinal deformity [9]. Over the last 50 years, spinal fusion has become the gold standard for the treatment of several degenerative spinal disorders. Despite the many benefits of fusion surgery, there are several complications associated with this technique, including adjacent segment degeneration and pseudoarthrosis [10, 11]. Biomechanical studies have shown that fusion surgeries cause increased motion loading, which increases the stress placed on adjacent vertebral segments, and long-term clinical studies have shown radiographic degenerations of the adjacent vertebral segments [12–14]. The incidence of adjacent segment degeneration after fusion surgeries is in the range of 5.2% to 100% [15]. Among the lumbar fusion surgery procedures, those performed between the thoracolumbar junction and the lumbosacral junction (the so-called “floating fusions”) appear to be associated with the greatest risk [14]. As a result, additional surgeries are often required to treat adjacent segment degenerations after lumbar fusion surgeries [16].

As mentioned above, motion preservation surgeries have been developed for the treatment of lumbar degenerative diseases in order to prevent adjacent segment degenerations [17, 18]. Sengupta described the hypothesis behind dynamic stabilizations, control abnormal motions, so that greater physiological load transmissions can relieve pain and prevent adjacent segment degenerations. A remote expectation is that once normal motion and load transmission is achieved, the damaged disc may repair itself, unless the degeneration is too advanced [19]. Posterior motion-sparing systems have been designed to off-load the posterior facets and annulus and to control motion in defined planes. By stabilizing vertebral motion, pain may be minimized, and the controlled motion may also decrease the secondary effects of fusion [20]. 

Posterior dynamic stabilization system devices can be classified into three types: (1) posterior interspinous spacers, (2) posterior pedicle fixation-based dynamic stabilization devices, and (3) total facet replacement devices [21]. Kaner et al. recently classified these dynamic systems [22], and the most important differences were seen in the groups where dynamic rods and screws were used together. This group was accepted as an independent group in their classification. In this paper, we will summarize and discuss the devices in which dynamic rods and screws are used together.

## 2. Posterior Pedicle Fixation-Based Dynamic Stabilization Devices

### 2.1. Dynamic Rods

#### 2.1.1. Graf Ligament

**Figure 1:**
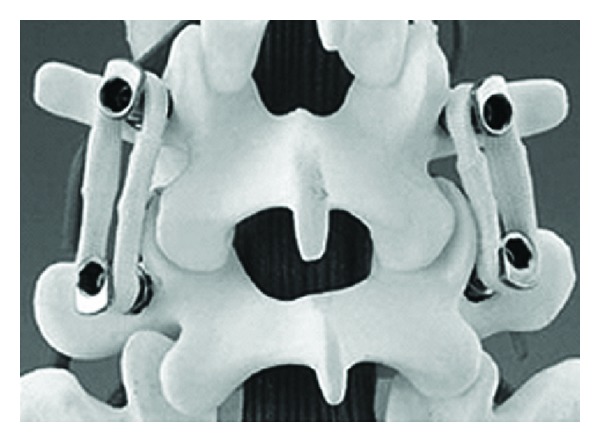
Graf ligamentoplasty system (left) and application on the lumbar spine model.

In 1992, Graf described the use of the Graf ligamentoplasty system to treat low back pain without fusion [23]. According to his theory, abnormal rotary motion was the primary source of mechanical low back pain. He later improved the Graf ligamentoplasty system by inserting titanium pedicle screw anchors into the vertebra, both superior and inferior to the symptomatic level, and using a braided polypropylene tension band to link the titanium pedicle screws (Figure 1).

Due to compressions on the posterior annulus, it was claimed that Graf's system allowed annular tears to heal. Initial outcomes from Graf ligamentoplasty surgeries showed only modest improvements in functional ability and required high rates of reoperation. Grevitt et al. reported on a study of 50 patients who underwent Graf ligamentoplasty for intractable, symptomatic degenerative disc disease and chronic low back pain [24]. The Oswestry disability index (ODI) improved postoperatively from 59% to 31%, but postoperative radiculopathies were reported in 12 of the 50 patients. Therefore, prophylactic foraminal decompressions prior to device placement were recommended. Markwalder and Wenger reported long-term results in 41 patients treated with Graf ligamentoplasty. Sixty-six percent of patients reported no pain, 25.7% of patients reported significantly less pain, and 7.7% of patients reported somewhat less pain. The authors concluded that in younger patients with painful mechanical spine disease refractive to conservative treatment, Graf ligamentoplasty is an acceptable alternative to fusion surgery and provides long-term symptomatic relief [25]. 

On the other hand, Hadlow et al. reported on a retrospective case-control comparison between Graf ligamentoplasty and posterolateral fusion in a series of 83 patients suffering from low back pain [26]. There was a significantly high rate of reoperation in Graf ligamentoplasty groups 2 years after surgery (72%). Therefore, for the treatment of low back pain, the authors concluded that Graf ligamentoplasty did not show superiority to posterolateral fusion. 

Graf ligamentoplasties also produce a significant increase in lateral canal stenosis, especially when patients exhibited preexisting degenerative changes in the facet joints or in the infolding of the ligamentum flavum, owing to the marked lordosis of the segment instrumented. Early clinical failures were associated with this surgical complication [24]. Graf ligaments transfer the load from the anterior aspect of the disc to the posterior annulus, thereby increasing the disc pressure in this region. This may explain the late failure of the Graf ligament, which accelerates disc degeneration by overloading the posterior part of the disc [19].

Recent randomized evaluations reported better clinical outcomes in patients that underwent Graf ligament placements versus fusions. If the patient is experiencing spondylolisthesis or flexion instability, then a Graf ligamentoplasty is a good choice. However, if the patient complains of scoliosis or lateral listhesis, then the Graf ligamentoplasty is not a good choice and could lead to a higher likelihood of reoperation.

#### 2.1.2. Dynamic Neutralization System (Dynesys)

**Figure 2:**
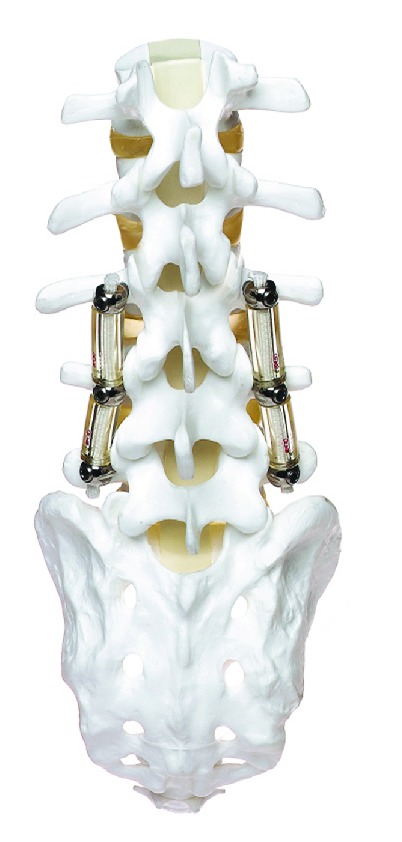
Dynesys device applied on a spinal model.

**Figure 3:**
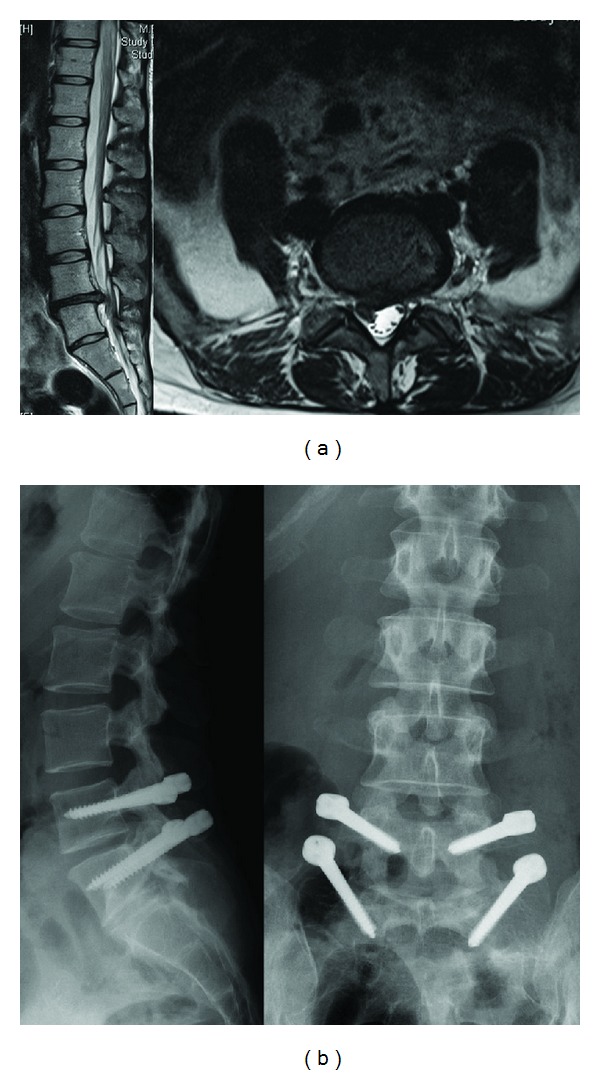
34-year-old female patient with right leg pain and back pain. (a) T2 weighted MRI scans show L4-5 disc herniation; (b) the Dynesys system was applied.

The dynamic neutralization system (Dynesys) was developed by Stoll et al. 2002 [27]. This system consists of titanium alloy (protasul 100) pedicle screws, polyester (sulene-PET) cords, and polycarbonate urethane (sulene-PCU) spacers (Figure 2). The PET cord resists tensile forces and provides resistance to spine flexion, similar to the concept used in Graf ligamentoplasties. However, the Dynesys PCU spacers resist compression during extension and thereby prevent foraminal narrowing by maintaining foraminal height and decreasing load to the posterior annulus [28, 29] ( Figure 3). 

The results from the clinical studies on the Dynesys system are twofold. Cheng et al. reported that there was no significant difference between using the Dynesys or using traditional rigid fusion to treat adjacent segment disease [10]. However, several studies have suggested that using the Dynesys as a nonfusion device results in superior clinical outcomes compared with traditional rigid fusions [27, 30, 31]. Grob et al. reported on a retrospective study of 50 patients treated with the Dynesys for either degenerative disc disease or stenosis-associated instability. Thirty-one of these patients had at least a 2-year followup period [32]. Back pain improved in 67% of the patients, 30% of the patients reported that their condition was unchanged, and 3% of the patients reported a worsening of symptoms. Leg pain improved in 64% of the patients, 21% of the patients reported that their condition was unchanged, and 14% of the patients reported an increase in pain after treatment. However, functional capacity only improved in 40% of the patients, and within the 2-year followup period, 6 of the 31 patients (19%) underwent an additional operation. 

Bothmann et al. evaluated clinical, radiographic, and computed tomography (CT) scans in 54 consecutive cases that underwent nonfusion surgery using the Dynesys [33]. Postoperative pain scores improved in 29 of the cases (79%), and scores were optimal when dynamic fusion was used in conjunction with nerve root decompression. The outcomes were not superior to the conventional rigid fusion system, and complications required revision surgery in 27.5% of the cases.

Cienciala et al. studied dynamic stabilizations with the Dynesys in 102 patients with degenerative disc diseases [34]. The improvement in the patients' health status was statistically significant during all 3-year postoperative periods. The Dynesys resulted in the postoperative disappearance of disc bulging and the restoration of both the posterior longitudinal ligament and the space in the lumbar spinal canal; repeated MRI examinations confirmed the disappearance of the bulge in these 26 patients. In their three-year followup period, patients had improved subjective feelings, morphological findings, pain, and functional status. 

Dynesys treatment is indicated for patients with degenerative diseases in the lumbar motion segment, instability, and in combination with functional or structural spinal canal stenosis. Contraindications for this system are spinal fractures, infections, lytic/isthmic spondylolisthesis, degenerative spondylolisthesis >I°-II°, facetectomy, and stabilization of the thoracic and cervical spine. 

#### 2.1.3. Accuflex Rod System

**Figure 4:**
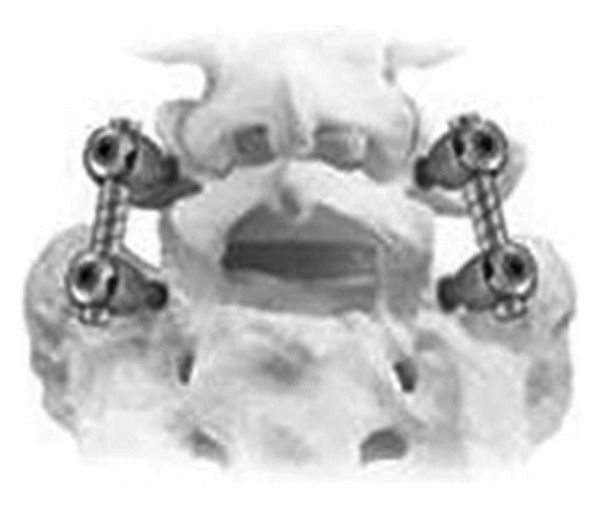
The Accuflex rod system.

The Accuflex rod system (Globus Medical Inc.) includes a dynamic rod and 6.5 mm pedicle screws made of titanium. The rod has double helical cuts that perform flexion-extension movements while providing a posterior tension band that can unload the disk (Figure 4). This system received FDA clearance for single level dynamic fusion. In a study conducted by Reyes-Sánchez et al., 20 consecutive patients underwent dynamic stabilization surgery with the Accuflex rod system to treat lumbar spinal stenosis and dysfunctional segment motion [35]; the clinical, radiographic, and magnetic resonance imaging (MRI) findings were fully described. During a 2-year followup period, 22.22% of the patients required device removal due to fatigue, while there was no progression of disk degeneration observed after implantation of the Accuflex system in 83% of the patients. Three patients (16%) also showed disc rehydration in followup MRI imaging. Even with a relatively high rate of device removal (22.22%), the use of the Accuflex rod system provided enhanced clinical benefits and stopped the degenerative process in 83% of the patients.

#### 2.1.4. Isobar TTL

**Figure 5:**
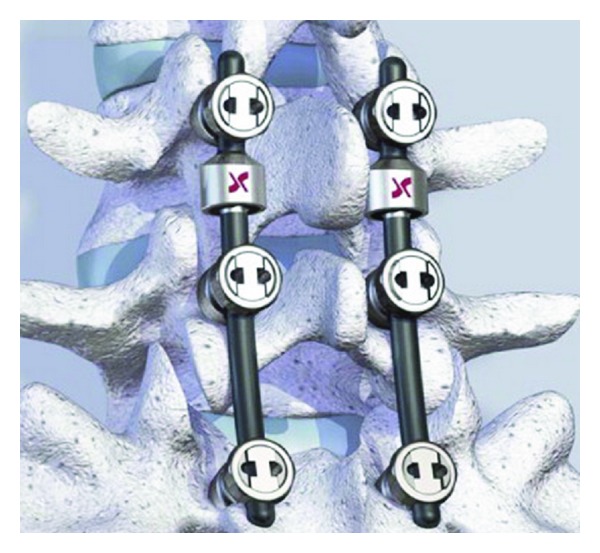
The Isobar TTL device.

The Isobar TTL system (Scient'x USA) is one of the first described semirigid rods. This implant received FDA clearance for use as an adjunct to spinal fusion in 1999. This system is composed of a titanium alloy rod with a dampener made of stacked titanium alloy o-rings. The Isobar TTL system allows a small amount of both axial and angular motion via this dampener (Figure 5). Perrin and Cristini reported on a retrospective study of 22 patients that underwent dynamic stabilization using the Isobar TTL system to treat lumbar spondylolisthesis [36]. The slipped levels were treated with polyetheretherketone (PEEK) cage, followed by a two level posterior fixation using the Isobar TTL system. During the 8.27-year followup period, 68.2% of the patients reported mild leg pain, 72% of the patients reported no or mild back pain, and 91% of the patients were complaint free. The adjacent level was also protected by the Isobar TTL system. 

#### 2.1.5. CD-Horizon Legacy PEEK Rod

**Figure 6:**
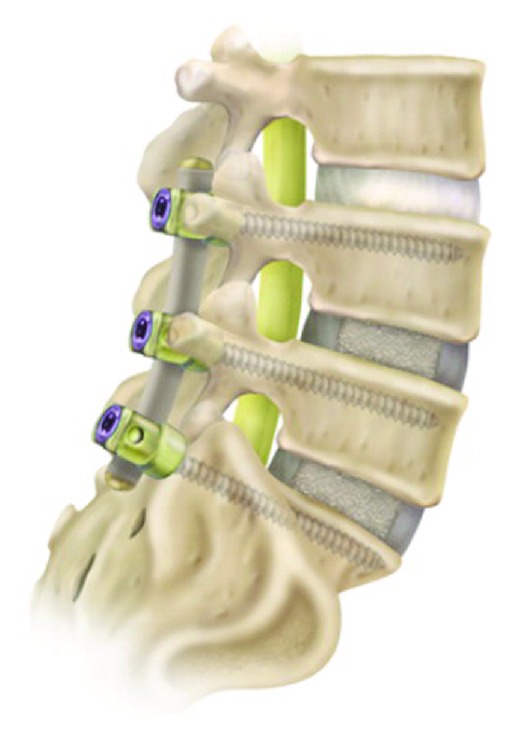
The CD-Horizon Legacy PEEK rod.

**Figure 7:**
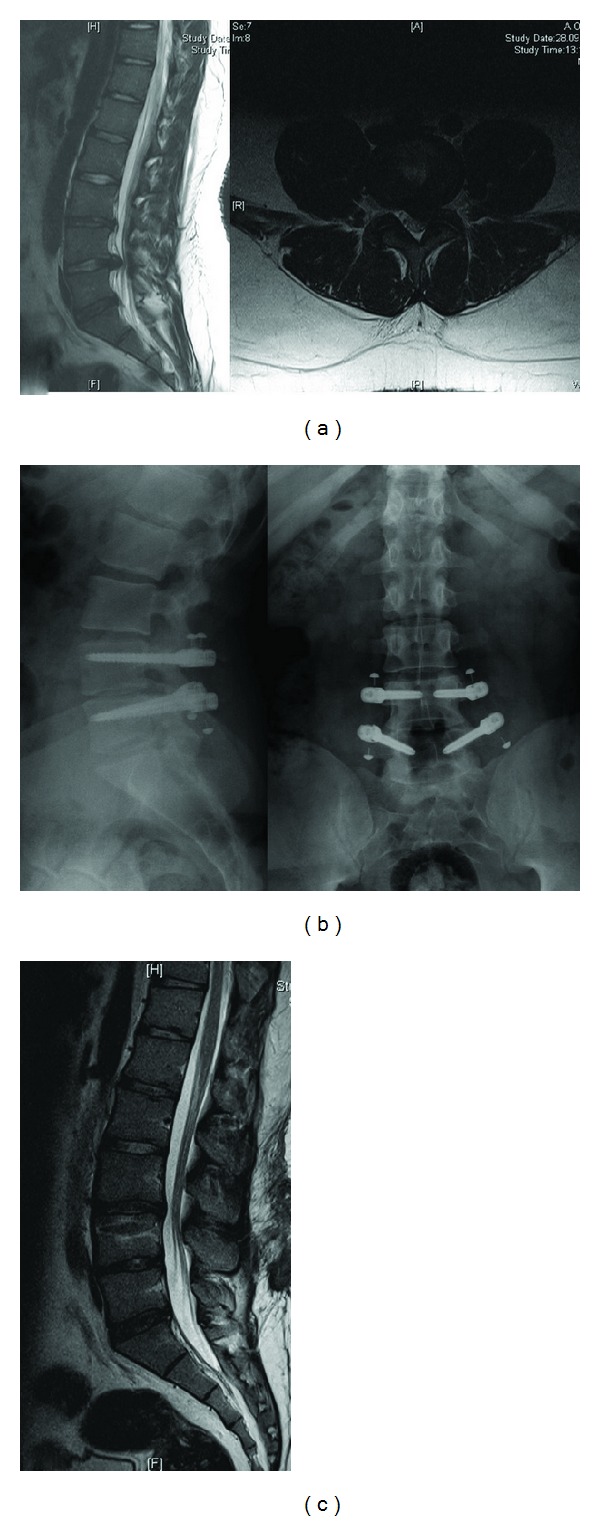
20-year-old male patient with left leg pain and back pain. (a) T2 weighted MRI scans show L4-5 disc herniation; (b) PEEK rod was applied; (c) the patient was in very good condition, and T2 weighted MRI scan showed a nearly normal appearance after one year.

The CD-Horizon Legacy PEEK rod (Medtronic Sofamor Danek, Memphis, TN) is composed of polyetheretherketone and is more flexible than the titanium rods (Figure 6). This system received FDA clearance in 2005. The PEEK rod is currently FDA approved to treat adjunct fixation for a one-level interbody fusion. Abode-Iyamah et al. reported a cadaveric study that measured intradiscal pressure differences between the PEEK rod and the titanium rod [37]. Pressure differences were greater for the titanium rods compared with the PEEK rods. However, it has not been determined whether dynamic rods, such as DYNESYS and Accuflex or PEEK rods could be used with dynamic screws instead of using rigid titanium rods because PEEK rods are more flexible compared with titanium rods (Figure 7). As a result, the authors concluded that the PEEK rods decreased adjacent disc disease by maintaining a lower intradiscal pressure.

#### 2.1.6. Bioflex Spring Rod Pedicle Screw System

**Figure 8:**
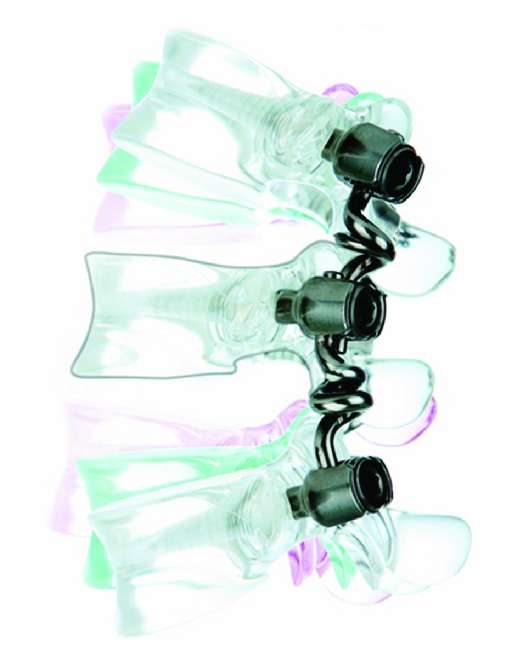
The Bioflex dynamic stabilization system.

The Bioflex system (Bio-Spine Inc.) is a pedicle screw-based system that is composed of rod-shaped Nitinol with one or two loops to confer stability in flexion, extension, and lateral bending (Figure 8). Nitinol is an alloy of titanium and nickel, also called the “memory metal” due to its ability to return to its original shape after deformation. In a study conducted by Kim et al., 103 patients treated with the Bioflex system were observed preoperatively and postoperatively for range of motion (ROM) changes. The patients were divided into two groups: dynamic stabilization with or without posterior lumbar interbody fusion (PLIF) (Group 1) and rigid fixation (PLIF+ Bioflex system only) (Group 2). The changes in the ROM in looped segments that were treated with PLIF were significantly reduced, but the changes in the ROM in looped segments without PLIF were not significant. The authors concluded that the Nitinol Bioflex dynamic stabilization system achieved stabilization while simultaneously permitting physiological movement which in turn decreases the degeneration of adjacent segments [38]. 

In a study conducted by Zhang et al., 12 patients were treated with the Bioflex system to examine functional motion one or more years after Bioflex system placement. Six patients were treated with a L3-4-5 construct, and another six patients were treated with a L4-5-S1 construct. The followup period varied from 12 to 33 months; standing neutral lateral flexion, extension, and posteroanterior radiographs were obtained at 3, 6, 9, 12, and more than 12 months postoperatively. The ROM for whole lumbar lordosis and segments from L2 to S1 were determined. The authors concluded that the Bioflex system was able to preserve functional motion to some degree at instrumented levels. However, although total lumbar lordosis was preserved, the ROM at the implanted segments was lower than their preoperative values [18].

#### 2.1.7. Fulcrum-Assisted Soft Stabilization System (FASS)

**Figure 9:**
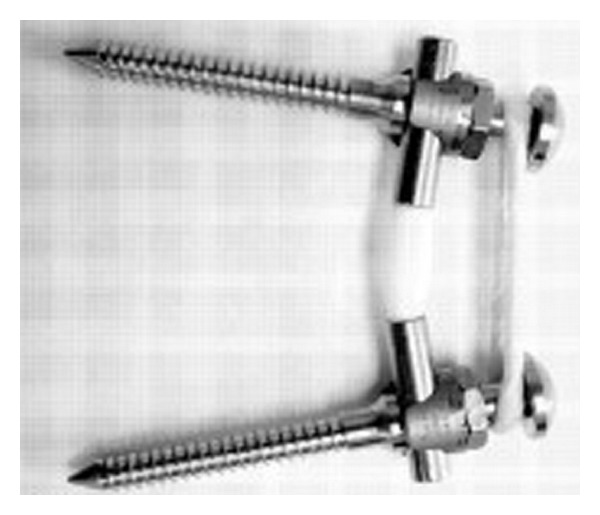
The Fulcrum-Assisted Soft Stabilization system (FASS).

The FASS (Fulcrum-Assisted Soft Stabilization) system was developed by Sengupta and Mulholland [39] to address the most common disadvantages of the Graf system (Figure 9).

1. Increased lordosis, which produces a narrowing of the lateral recess, leading to root entrapment, especially with preexisting facet arthropathy.
2. Increased loading of the posterior annulus, which is typically observed in patients with painful degenerated discs.

The presence of a fulcrum may prevent both of these problems. The fulcrum is placed between the pedicle screws, in front of the ligament, and acts by distracting the posterior annulus. The elastic ligament is placed at the heads of the pedicle screws, posterior to the fulcrum and maintains lordosis. The fulcrum transforms the compressive effect of the elastic ligament into an anterior distraction force that unloads the disc. 

### 2.2. Dynamic Screw

#### 2.2.1. Cosmic Posterior Dynamic System

**Figure 10:**
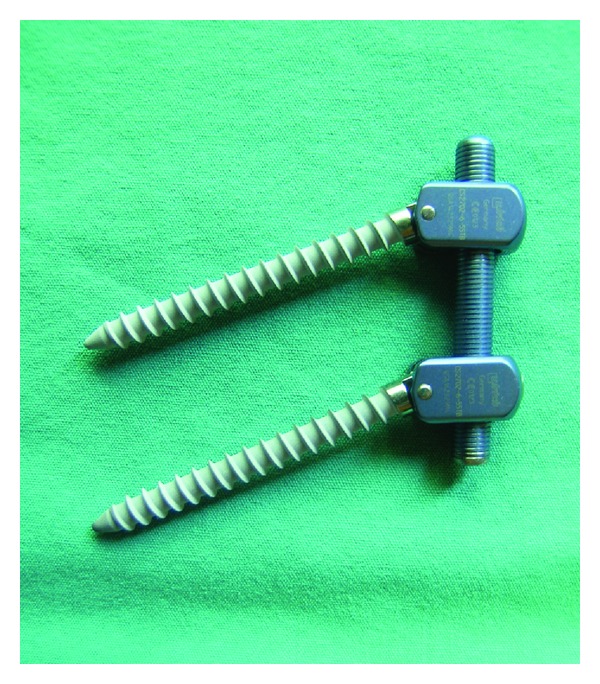
The Cosmic posterior dynamic system.

The Cosmic posterior dynamic system (Ulrich medical) is a pedicle screw-based dynamic stabilization system (Figure 10). Indications for this device include spinal stenosis, degenerative spondylolisthesis. The main characteristic of this system is a hinged pedicle screw head that allows segmental motion, thereby reducing stress at the bone-screw interface. The screw threads are coated with calcium phosphate to promote ingrowths and assist in long-term fixation. 

**Figure 11:**
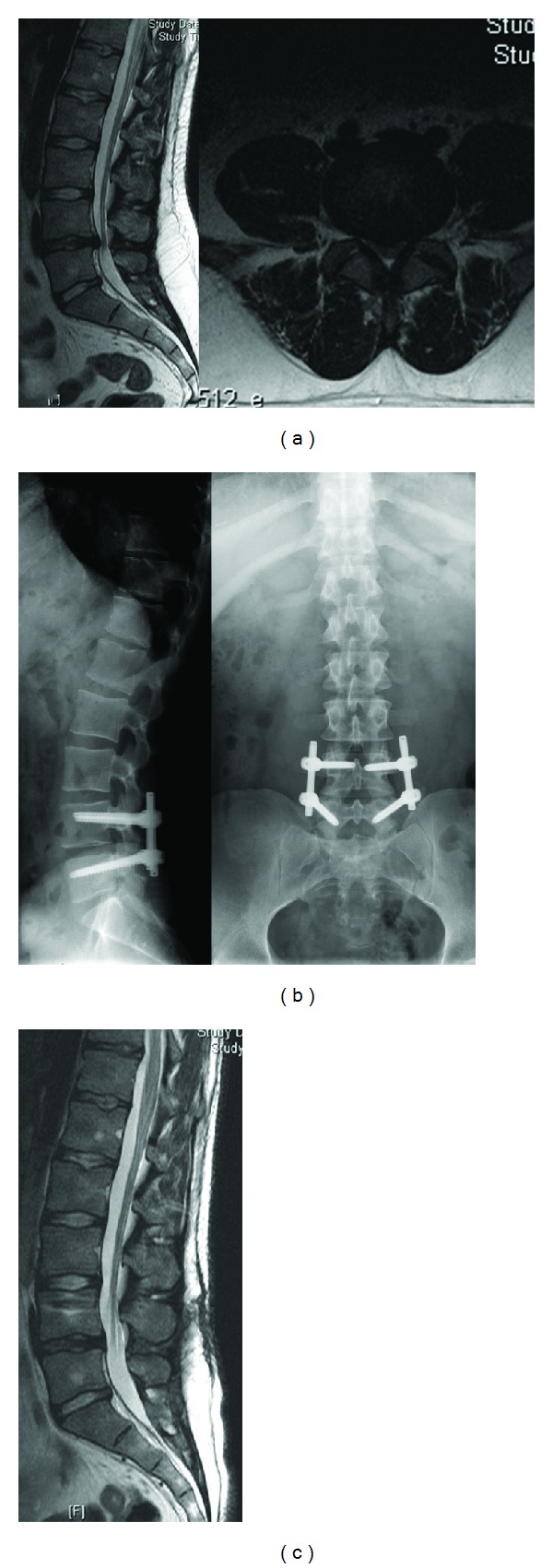
24-year-old male university student, with acute attacks of low back pain (2–4 times per year). He experienced right leg pain in a sciatalgia form, accompanied by severe low back pain in the last attack. In his neurological examination, he had L5 hypoesthesia but no motor deficit. (a) T2 weighted MRI scans show l4-5 disc herniation; (b) cosmic was applied; (c) the patient was in very good condition, and T2 weighted MRI scan shows a nearly normal appearance after one year.

A hinged screw stabilizes the spine in a nearly rigid system [40, 41]. The results are similar to fusion after two years of followup [42–44]. Kaner et al. found that treatment with hinged screws was effective for addressing degenerative spondylolisthesis, spinal canal stenosis [43, 45], and recurrent disc herniations [46]. Similar results were found after a multilevel study of dynamic screws [47] (Figure 11).

Von Strempel et al. reported on a two year followup study for patients surgically treated with the Cosmic system to relieve degenerative lumbar disease [48]. The results of this study showed that the Cosmic system is an alternative to traditional fusion surgery to treat degenerative lumbar disease, but long-term followup studies are still necessary to fully evaluate this system on adjacent-level diseases. 

Stoffel et al. published results from a study of 103 patients that were consecutively treated using the Cosmic system for painful degenerative segmental instability ± spinal stenosis between April 2006 and December 2007 [49]. This study showed that dynamic stabilization with Cosmic achieved significant improvements in pain, related disability, mental/physical health, and mobility, respectively, and a high rate of satisfied patients. 

#### 2.2.2. Saphinas System

**Figure 12:**
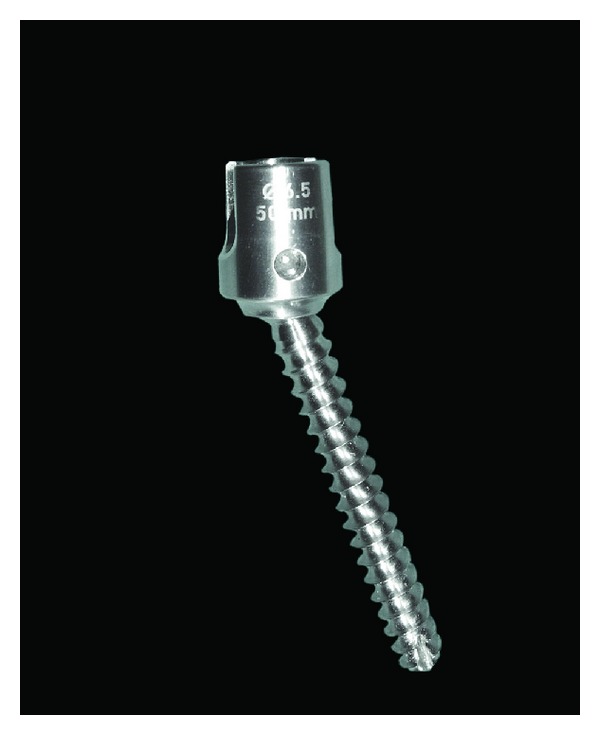
Saphinas screw.

**Figure 13:**
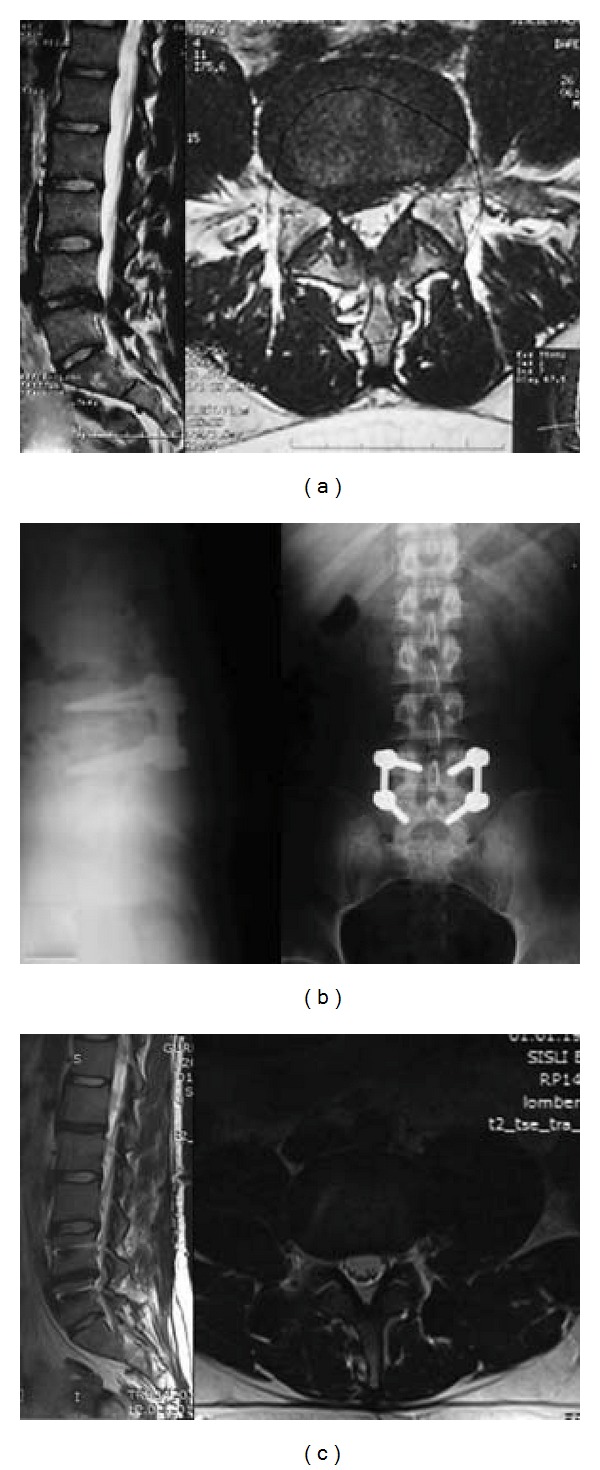
18-year-old, male patient with severe acute back pain attacks. In the last attack, he became aware of sciatalgia on the right side. He had no response to medical treatment and physical therapy; he had difficulties in daily life. (a) Preoperative MRI showed posterior annular defects, and a bulging disc to the right L5 nerve root is compressed; (b) saphinas system was applied; (c) one year after surgery the patient is very satisfied with this surgery, and MRI showed no bulging or defects of posterior annulus.

The Saphinas system (Medikon Company) is another treatment which localizes between the head and the body of the screw (Figure 12). This treatment provides flexion extension movements and 1° of rotational movement past the designed screw. Biomechanical studies have shown that this system demonstrates sufficient stabilization over degenerative motion segments [40]. Clinical studies have also shown that the Saphinas system established a stable, rigid system [44] (Figure 13).

### 2.3. Dynamic Rods with Dynamic Screws

The main function of dynamic rods is to provide enough posterior tension over the posterior column of the spine. In biomechanical studies, the dynamic rods acted as a rigid system, and their stiffness was too close to the rigid system [50]. Dynamic rods are more flexible than the well-known rods currently available. Biomechanical studies show that more flexible rods with dynamic screws can stabilize the spine more effectively [50]. 

**Figure 14:**
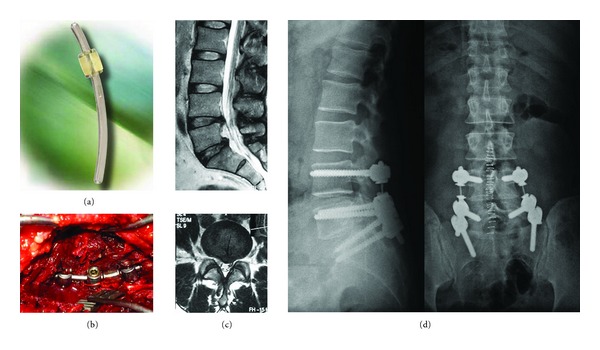
((a) and (b)) Agile rod; (c) 40-year-old male patient with severe right leg pain; (d) agile rod was applied, and the patient was in very good condition in postoperative year one.

**Figure 15:**
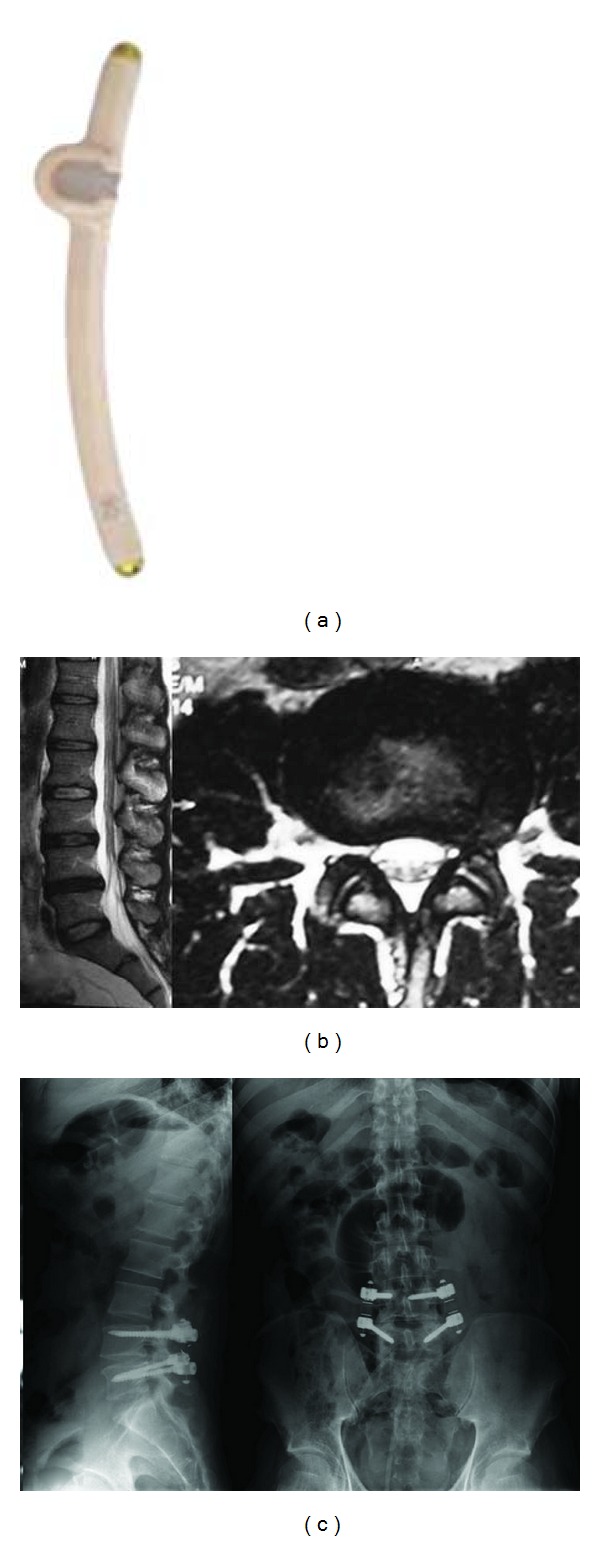
(a) BalanC rod. (b) 25-year-old male patient with severe back pain attacks every two months in last year. T2 weighted MRI scans show posterolateral annular rupture on the left side. (c) After repairing the posterior annulus under a microscope, dynamic screws (Cosmic screw) were used with dynamic rods (BalanC). The patient was very well 6 months after the of operation.

An agile rod is the first rod that we used with dynamic screws (Figure 14) [51]. However, agile rods were withdrawn from the market after a more flexible rod, BalanC, was developed and used with dynamic screws. Our preliminary results are very promising, and upon the study's completion, our results will be published (Figure 15). 

### 2.4. The Systems Represent Facet Functions

#### 2.4.1. Stabilimax NZ

**Figure 16:**
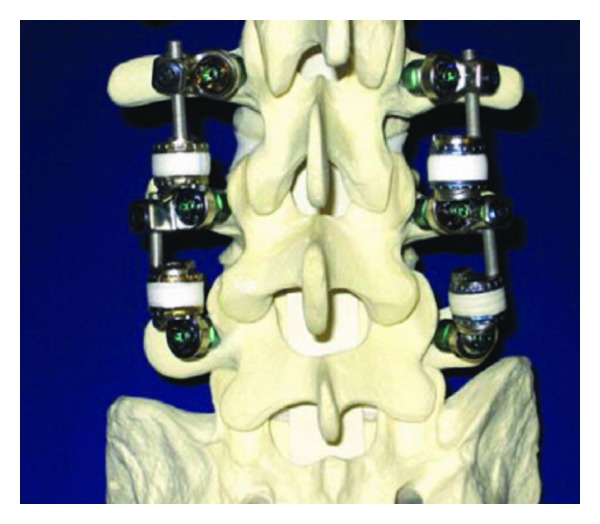
Stabilimax NZ device.

Stabilimax NZ (Applied Spine Technologies, New Haven, CT) is a pedicle screw-based posterior stabilization system that was designed as an alternative to fusion treatment to treat low back pain (Figure 16). Panjabi reported on the importance and the role of the “neutral zone” (NZ) in the development of spinal instability [3]. The NZ is a region of intervertebral motion around the neutral posture where little resistance is offered by the passive spinal column. It is believed that the NZ increases during disc degeneration and injury, resulting in greater instability and pain. The Stabilimax NZ system was developed to reduce the impact of the NZ on mechanical back pain. The Stabilimax NZ system is composed of a rod with dual concentric springs that maintain the spinal segment in a neutral position during spinal motion. 

This system received FDA/IDE approval to begin randomized controlled clinical trials comparing instrumented fusion to Stabilimax for the treatment of spinal stenosis with or without grade I spondylolisthesis. The data from these studies have yet to be published.

#### 2.4.2. Dynamic Stabilization System (DSS)

**Figure 17:**
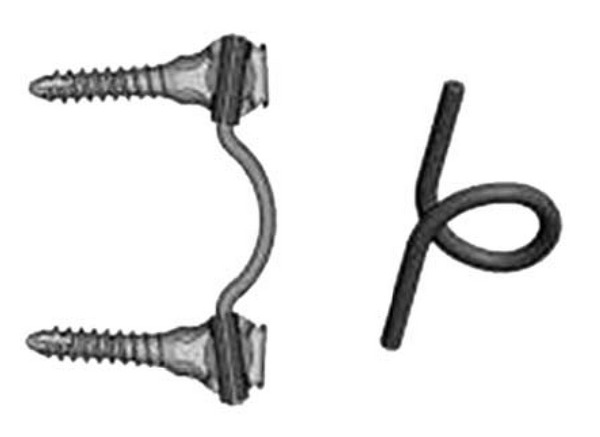
The DSS-I system.

The DSS system was developed by Sengupta et al. [52] as an improvement of the FASS system. Biomechanical studies show that the FASS system produces too much loading during flexion which leads to early device failures. The DSS system has two designs that have been tested in the laboratory. The DSS-I consists of a titanium spring, made of a 3 mm cross-section diameter of spring-grade titanium wire (Figure 17). The DSS-II system consists of an elliptical coil spring, made from 4 mm spring-grade titanium rods. 

In 2006, Sengupta et al. reported the results of a 16 patient study where participants were treated with the DSS for single level mechanical back pain associated with disc degenerations with a two-year followup period [53]. The mean ODI scores decreased from 65% to 27%, and VAS scores decreased from 7.3 to 3.7. There were no reports of instrumentation failure or screw loosening.

### 2.5. Total Facet Replacement Devices

#### 2.5.1. Total Posterior Arthroplasty System

**Figure 18:**
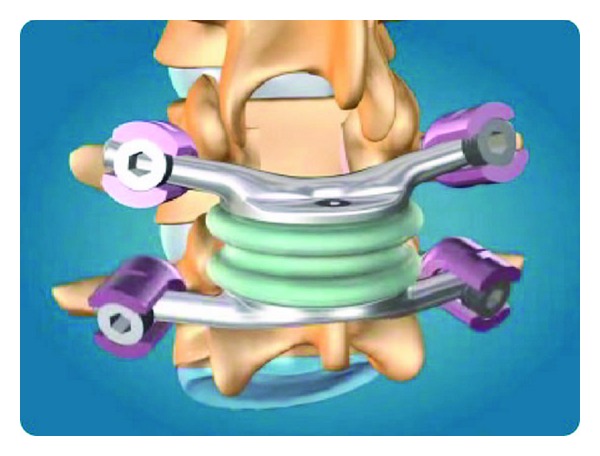
Total Posterior Arthroplasty System.

The Total Posterior Arthroplasty System (TOPS) uses a pedicle screw-based posterior arthroplasty prosthesis that was developed to provide dynamic, multiaxial, and 3-column stabilization while preserving normal motion (Figure 18). 

Wilke et al. published the results of an in vitro study using the TOPS in six human cadavers [54]. The cadavers were loaded with pure moments of ±7.5 Nm in flexion/extension, lateral bending, and axial rotation. The following states were investigated: (1) intact; (2) after bilateral laminectomy, including facetectomy of the lower facet joints, of the upper vertebra L4; and (3) after device implantation. The ROM, neutral zone, and intradiscal pressure were determined from a third round of treatment. In a second step, the ROM during axial rotations was determined as a function of the different flexion/extension postures. The authors concluded that the TOPS implant almost ideally restored the ROM in lateral bending and axial rotation compared to that of the intact specimen.

McAfee et al. reported the results of a study in which 29 patients were treated with the TOPS for spinal stenosis and/or spondylolisthesis at L4-5 due to facet arthropathy [55]. The average surgery lasted 3.1 hours, and the patients' clinical status improved significantly following treatment with the TOPS device. One year after surgery the mean ODI score decreased by 41%, and the 100-mm VAS score decreased by 76 mm. Radiographic analysis showed that lumbar motion was maintained, disc height was preserved, and there was no evidence of screw loosening; there were no device malfunctions, no migrations, and no device-related adverse events reported during the study.

#### 2.5.2. Total Facet Arthroplasty System

**Figure 19:**
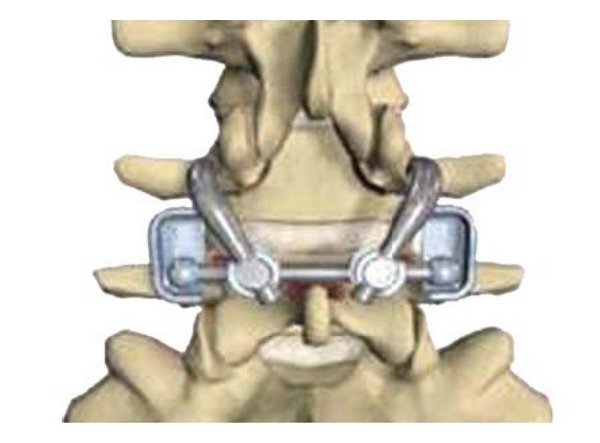
Total Facet Arthroplasty System.

The Total Facet Arthroplasty System (TFAS) is a posterior nonfusion stabilization device designed to stabilize the spine after a laminectomy to treat moderate-to-severe spinal stenosis (Figure 19). The TFAS is designed to replace the degenerated facet joints with prosthetic metal joints, as used in knee and hip arthroplasty.

Phillips et al. reported an in vitro study using TFAS in nine human cadaveric spine specimens [56]. Nine human lumbar spines (L1 to sacrum) were tested inflexion-extension (+8 to −6 Nm), later albending (±6 Nm), and axial rotation (±5 Nm). Flexion-extension was tested under 400 N following preload. Specimens were tested intact, after complete L3 laminectomy with L3-L4 facetectomy, after L3-L4 pedicle screw fixation, and after L3-L4 TFAS implantation. The ROM was assessed in all of the tested directions. The neutral zone and stiffness during flexion-extension were calculated to assess the quality of motion. The authors concluded that after a wide range of decompressions on the neural elements, TFAS overcame the need for fusion by stabilizing the surgically modified spine in a manner similar to intact vertebrae, while restoring the physiologic kinematics (range and pattern of motion) at an operative level. Furthermore, TFAS resulted in more natural kinematics in the adjacent levels when compared with fusion.

### 2.6. Posterior Interspinous Spacers

#### 2.6.1. Wallis Implant

**Figure 20:**
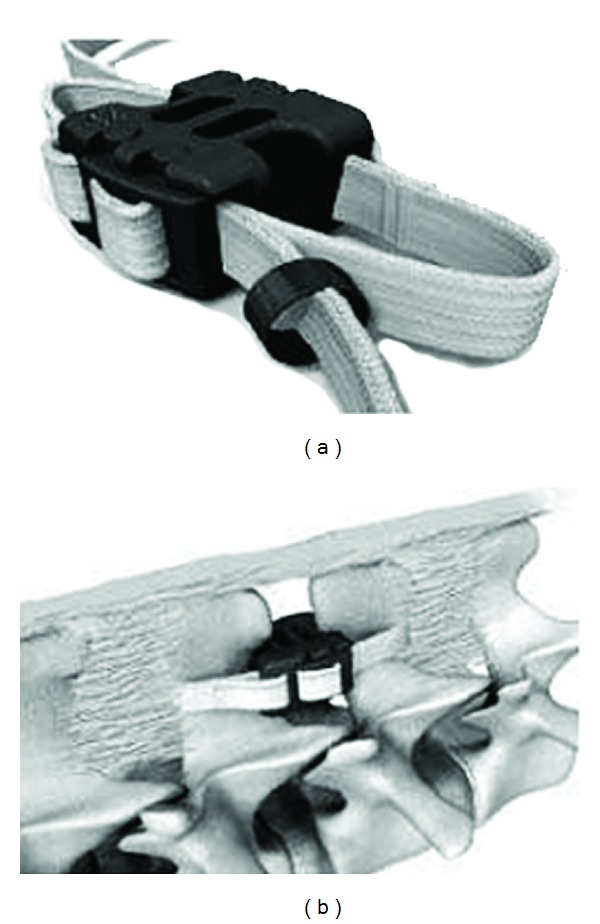
The Wallis implant.

Senegas et al. described an interspinous spacer in 1988 [57]. This device was made of titanium and held between the spinous processes via a dacron tape. After the successes of the first implant over three hundred patients, the authors redesigned the system known as “Wallis implant” which uses PEEK (polyetheretherketone) material as the spacer instead of titanium (Figure 20). The interspinous implant, located the interspinous space, blocks the extension of segment, and via distraction of the spinous processes, provides a relative flexion posture that known as a posture relieving neurogenic claudication pain by enhancing foraminal width. Additionally, the Dacron tape acts as a flexion limitation factor at the implant's located segment. Because of these features, this device can be described as a hybrid of interspinous distraction device and interspinous ligament. The authors recommend the usage of the Wallis system in the following indications: (1) discectomy of massive herniated disc leading to substantial loss of disc material, (2) a second discectomy for recurrence of herniated disc, (3) discectomy for herniation of a transitional disc with sacralization of L5, (4) degenerative disc disease at a level adjacent to a previous fusion, and (5) isolated Modic 1 lesion leading to chronic low-back pain.

#### 2.6.2. X-Stop

**Figure 21:**
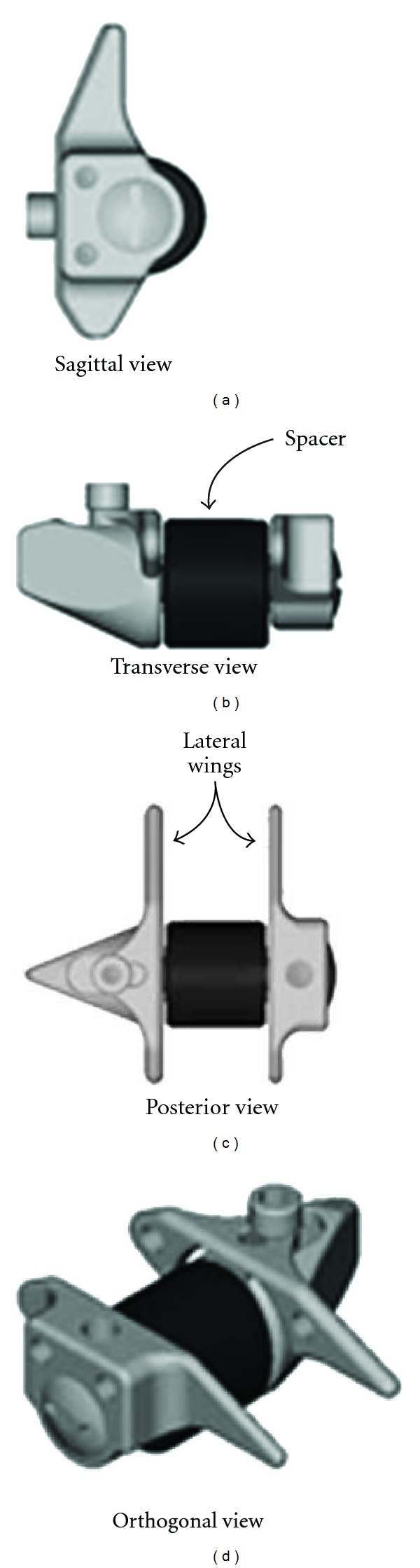
A sagittal, transverse, posterior, and orthogonal view of X-Stop implant.

This titanium interspinous distraction device (Figure 21) (X-Stop, St. Francis Medical Technologies, Inc., Alameda, CA) has been introduced as a minimal invasive surgical procedure to treat symptomatic degenerative lumbar spinal stenosis. This device can be introduced by a minimally invasive approach under local anesthesia and may be useful for treatment of degenerative lumbar spinal stenosis in elderly patients who cannot take general anesthesia because of comorbid conditions. There are many controversial researches in the literature about clinical results of X-Stop device. While Verhoof et al. reported that X-Stop interspinous distraction device showed an extremely high failure rate defined as surgical reintervention after short-term followup in patients with spinal stenosis caused by degenerative spondylolisthesis [58]. Zucherman et al. reported that X-Stop offers a safe and effective treatment for lumbar spinal stenosis [59].

## 3. Conclusion

Traditional fusion surgeries have been performed for several years, as the predominant technique used to treat degenerative spinal disorders. Although there are several benefits to use this surgical technique, adjacent segment diseases occur due to a transfer of stress from a stabilized motion segment to the adjacent level. Dynamic stabilization of the spine was developed to solve this problem by mimicking natural spine movements. Transferring the load from a degenerated disc or facet to a dynamic stabilization construct, while preserving segmental motion, is a critical feature required to develop novel dynamic stabilization devices. 

Short-term results from studies using these devices are promising, and the most common problem results from loosening failures because there is not enough active fusion mass to resist the physiological loads. Therefore, dynamic stabilization devices are not viable options to treat osteoporotic patients.
